# Beyond Aging: The Relationship Between Gerotranscendence and Oral Health‐Related Quality of Life—A Scoping Review

**DOI:** 10.1155/ijod/3541650

**Published:** 2026-10-13

**Authors:** Mithun B. H. Pai, Shweta Yellapurkar, Sampada Suresh Kulkarni, Shushma B. Rao, Kumar Gaurav Chhabra

**Affiliations:** ^1^ Department of Public Health Dentistry Manipal College of Dental Sciences Mangalore Manipal Academy of Higher Education Manipal India manipal.edu; ^2^ Department of Oral Pathology and Microbiology Manipal College of Dental Sciences Mangalore Manipal Academy of Higher Education Manipal India manipal.edu; ^3^ Department of Public Health Dentistry Srinivas Institute of Dental Sciences Rajiv Gandhi Institute of Health Sciences Karnataka Mangalore India srinivasgroup.com; ^4^ Department of Public Health Dentistry Nims Dental College and Hospital Nims University Rajasthan Jaipur India

## Abstract

**Background:**

“Aging India” is an important public health issue given the various multifactorial physical, psychological, and social aspects of old age. In 2000, the elderly population (≥60 years) in India accounted for 7.7% (77 million) of the total population; this percentage increased to 8.6% (104 million) in 2011 and increased again to 9.4% (125 million) in 2017. Oral health‐related quality of life (OHRQoL) is a multidimensional construct that impacts every aspect of daily activities. Gerotranscendence has been suggested to be an ability that enables adaptation and coping strategies from despair, found in Erikson’s eighth developmental task. The study aimed to systematically map and synthesize the existing literature on the relationship between gerotranscendence and OHRQoL among older adults and map any evidence gap on the topic.

**Methodology:**

This scoping review was conducted using Arksey O’Malley’s framework, reported in accordance with the Preferred Reporting Items for Systematic Reviews and Meta‐Analyses (PRISMA) ScR guidelines. The research question for this scoping review was: “What is the available evidence on the relation between gerotranscendence and oral health‐related quality of life (OHRQoL)?” The databases searched were PubMed, Embase, Scopus, and Web of Science. Grey literature was not included in this scoping review because it was poorly indexed and unstructured, and the effort required to structure it outweighed the benefits of rapid scoping. The search was conducted by one researcher, with the last search carried out in November 2025.

**Results:**

The search of various databases yielded 95 records. After removing the duplicates and completing the title abstract screening, a total of six articles were identified for the full‐text review. Based on the inclusion and exclusion criteria, four articles were excluded, and two were included in this scoping review; both were cross‐sectional studies and were conducted on a total of 2369 participants aged between 60–81 years.

**Conclusion:**

This scoping review shows an intersection between psychosocial aging theory and oral health research. Gerotranscendence seems to modulate the way older adults perceive and respond to challenges presented by their oral health, which suggests that well‐being later in life is not explained by clinical parameters alone.

## 1. Introduction

Worldwide demographic trends show a rapid growth in the aging population. The World Health Organization (WHO) predicted in 2015 that the share of individuals 60 years or older will nearly double to 22% of the total population by 2050 from only 12%. In absolute numbers, the number of individuals 60 years or older will grow from around 1 billion in 2020 to 2.1 billion by 2050 [1].

“Aging India” is an important public health issue given the various multifactorial physical, psychological, and social aspects of old age. In 2000, the elderly population (≥60 years) in India constituted 7.7% (77 million) of the overall population; this percentage increased to 8.6% (104 million) in 2011 and increased again to 9.4% (125 million) in 2017. Technological advances in healthcare, in combination with ongoing epidemiological change, are likely to accelerate the transition, with nearly 19% (324 million) of India’s population projected to be older adults by 2050. The state‐wise analyses also demonstrate considerable growth in the number of elderly people. In the Report of the Technical Group on Population Projections for India and States, 2011–2036 (National Statistical Office, Ministry of Statistics and Programme Implementation, Government of India, 2021), India experienced a rise of nearly 34 million elderly persons in 2021, relative to the 2011 censuses. This figure is projected to increase again by 56 million elderly persons by 2031. This clearly shows the high level of population aging that is occurring in the country [2].

Age is a key factor in mental health, with old age representing a key stage of transformation. Aging is not simply about physiological decline but the accompaniment of major challenges to psychological and social well‐being. Having multiple comorbidities, experiencing loneliness and social isolation, and having diminished personal and financial autonomy all contribute to the increased risk of psychiatric disorders in the elderly [3].

Tornstam proposed gerotranscendence, a psychosocial theory of aging, that expands upon Erikson’s developmental theory of psychosocial development to describe a qualitative effect in perspective during late life. Gerotranscendence emphasizes a growing connectedness with the universe and previous generations (cosmic), development of ego integrity and reduced self‐centeredness (coherence), and selective involvement with an increased desire for solitude (solitude). Gerotranscendence proposes that aging is a positive and adaptive process that may enhance coping capabilities, acceptance, and overall well‐being in older adults; it may protect against late‐life psychological incidents (anxiety and depression) through several interconnected psychospiritual mechanisms. The cosmic dimension involves a transformed perception of time, a stronger sense of connection with past and future generations, greater acceptance of life’s finiteness, and reduced apprehension of death. These changes may lessen existential anxiety and help older adults interpret aging, loss, and mortality as meaningful aspects of the life course [4–6].

Oral health‐related quality of life (OHRQoL) is a multilayered construct that impacts every aspect of daily activities. Studies have frequently documented poor oral health status among the elderly, and it has been suggested that oral health is deeply linked to a variety of systemic diseases. As a result, OHRQoL is a measure increasingly used to assess oral treatment needs, oral health, and the effects of dental treatment. Because OHRQoL affects patients’ expectations, experiences, and sociocultural backgrounds [7], it must be seen as an essential component of the overall health‐related quality of life. Gerotranscendence has been suggested to be an ability that enables adaptation and coping strategies from despair, found in Erikson’s eighth developmental task [8].

### 1.1. Aims/Objectives

• To systematically map and synthesize the existing literature on the relationship between gerotranscendence and OHRQoL among older adults.
• To identify the theoretical frameworks, measurement tools, and key findings in studies exploring gerotranscendence and OHRQoL.
• To highlight gaps in the current knowledge and suggest directions for future research and clinical practice in geriatric oral health and psychosocial well‐being.

## 2. Methodology

This scoping review was conducted using Arksey O’Malley’s framework [9] and reported in accordance with the PRISMA (Preferred Reporting Items for Systematic Reviews and Meta‐Analyses) ScR guidelines [10]. The research question for this scoping review was: “What is the available evidence on the relation between gerotranscendence and oral health related quality of life (OHRQoL)?”

### 2.1. Information Sources

A search strategy from the literature was formed using medical subject headings (MeSH) and text words related to gerotranscendence and OHRQoL. The databases searched were PubMed, Embase, Scopus, and Web of Science. Gray literature was not included in this scoping review as it was poorly indexed and unstructured, the structuring of which outweighs the benefit of rapid scoping, and the last search was carried out in November 2025.

### 2.2. Search Strategy

The keywords in the search strategy were related to gerotranscendence, older adults, and OHRQoL. These search terms were adapted for use in each database. Here is an example of the search strategy derived from one database:

(“quality of life”[MeSH Terms] OR “oral health related quality of life”[Text Word]) AND “gerotranscendence”[All Fields] AND (“aged”[MeSH Terms] OR “older adults”[Text Word]).

### 2.3. Eligibility Criteria

In this scoping review, the eligibility criteria for inclusion and exclusion of studies were based on the population, concept, and context (PCC) framework, which is the standard framework followed by the authors in their earlier scoping review publications [11].

#### 2.3.1. Population

Studies that were conducted on older adults above 60 years of age who were either dependent or independent of care were included. In this review, studies on older adults in support of palliative care or at the end of life were excluded.

#### 2.3.2. Concept

Studies on gerotranscendence and OHRQoL were included. Studies that were based on general health, quality of life, or psychological‐related studies were excluded.

#### 2.3.3. Context

The context of this review was a global perspective. Studies in which participants resided in communities, nursing homes, old‐age homes, long‐term facilities, or facilities housing older adults were included in this review, and studies on older adults in palliative care homes were excluded.

Articles published in English, which were original research and with full‐text availability, were considered. Review articles, letters to the editor, editorials, and articles without full‐text access were excluded [11].

### 2.4. Study Selection

Mendeley software version 2.77.0 from Mendeley Ltd. was used to manage the search results obtained [12]. Two reviewers, Mithun Pai B. H. and Shushma Rao B., independently screened the titles and abstracts according to the predefined eligibility criteria. Differences were resolved by consensus or with a third author’s input (Shweta Yellapurkar).

### 2.5. Data Extraction

The reviewers tabulated the data independently, discussed the results, and revised the extraction table in an iterative process. Any doubts or disagreements were deliberated on and resolved among the authors.

### 2.6. Synthesis of Results

To understand the effect of gerotranscendence on OHRQoL among older adults, the selected articles were subjected to analysis via ATLAS.ti 24.1.0 for Mac [13]. This method consisted of repeated readings of the final articles, and significant segments were identified, which were then coded by two authors (Mithun Pai B. H. and Shushma Rao B.). These codes were reevaluated to be refined and to establish main themes through subsequent discussions among the authors. The themes and their related codes were then represented in tabular form to expedite a comprehensive understanding of the outcomes from this review.

## 3. Results

The search of various databases yielded 95 records. After removing the duplicates (*n* = 11) and completing the title abstract screening, a total of six articles were selected for the full‐text review. Depending on the inclusion and exclusion criteria, four articles were excluded, and two were included in this review. This selection process is represented in the PRISMA flow diagram (Figure 1).

**Figure 1:**
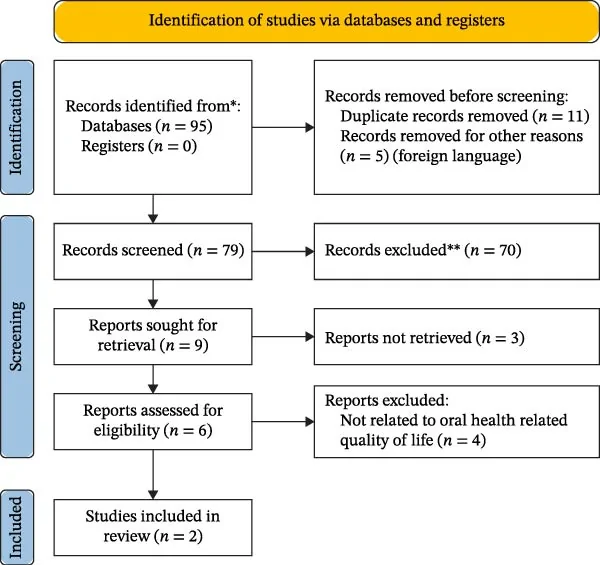
PRISMA flow diagram representing the process of article selection. The  ^∗^ indicates from the database, and  ^∗∗^ indicates nonrelevant/not related.

Figure 1 PRISMA flow diagram representing the process of arriving at the final articles selected for this review. The data extracted included the authors, year of publication, journal of publication, title of the studies, aim/purpose, study design, study setting, sample size, methodology, outcome, and conclusion. The final data extraction table is provided in Table 1.

**Table 1:** Data extraction table representing various characteristics of the included articles.

Author, year and journal	Title of study	Aim/purpose	Study design	Setting/sample size	Methodology	Outcome	Conclusion
Das et al., 2023 [8] The Journal of Indian Prosthodontic Society	Correlation between gerotranscendence and oral health‐related quality of life among elderly population in Davanagere city: A cross‐sectional survey	To assess the relationship between oral health‐related quality of life (OHRQoL) and gerotranscendence among elderly subjects in Davangere city.	Cross‐sectional Survey	Real‐world setting in Davangere 400	A self‐designed proforma was used to collect data. It contained both open‐ and closed‐ended questions. Data regarding demographic features, medical ailments and medications, habits, utilization of dental services, self‐perceived oral health, and Mini‐Nutritional Assessment—Short Form Index, Gerotranscendence Scale Type 2 questionnaire, Geriatric Oral Health Assessment Index (GOHAI), and oral health status clinically were recorded. The investigator carried out an oral examination using a CPI probe and mouth mirror.	The significant (*p* < 0.05) predictors of GOHAI were age, sex, marital status, nutritional level, missing teeth, socioeconomic status, self‐perceived oral health and self‐perceived need for treatment	OHRQoL was significantly negatively correlated with gerotranscendence. Gerotranscendence is an important confounder during assessing the association between several factors with OHRQoL.
Mihara et al., 2018 [14] Journal of Oral Rehabilitation	Relationship between gerotranscendence and oral health‐related quality of life	The purpose was to investigate the relationship between gerotranscendence and OHRQoL	Cross‐sectional study	For four geographic areas in both regions, urban and rural areas were included.	GOHAI was used to measure OHRQoL. Occlusal force was assessed with pressure‐sensitive sheets. Gerotranscendence was evaluated using the Japanese Gerotranscendence Scale Revised (JGS‐R).	On regression analysis GOHAI score was significantly associated with occlusal force, number of teeth, financial status and JGS‐R score.	There was significant association between GOHAI and JGS‐R scores, indicating high gerotranscendence score is seen with high GOHAI score. There is significant correlation between OHR‐QoL and degree of gerotranscendence.

Both articles included were cross‐sectional studies and were conducted on a total of 2369 participants aged between 60–81 years. Table 2 shows the age distribution and mean scores for gerotranscendence and OHRQoL (measured using the GOHAI) in both studies.

**Table 2:** The age distribution and mean scores of gerotranscendence and GOHAI in the included studies.

Study	Age (*n*)	Gerotranscendence score (mean)	GOHAI (mean)
Mihara et al. [14]	69–71 (999) 79–81 (970)	51.4	50.4
Das et al. [8]	60–75 (268) 76–90 (132)	19.5	41.3

The Japanese study [14] used the Japanese Gerotranscendence Scale‐Revised (JGS‐R). The Indian study [8] relied on the Gerotranscendence Scale Type 2. This shows how the tools reflect cultural adaptations of Tornstam’s original instrument [15]. The similarity in using recognized gerotranscendence scales supports a comparable understanding. However, it also suggests that more cultural validation is needed to address the specific psychosocial aspects of aging in different regions.

Both studies evaluated OHRQoL with the GOHAI, but Mihara et al. [14] added objective measures of occlusal force and number of teeth. The results indicated significant associations between the gerotranscendence scores, occlusal strength, and financial status. Das et al. [8] used subjective self‐assessments in combination with clinical examinations using CPI probes. Predictors of OHRQoL included age, gender, marital status, and nutritional status as key covariates. Hence, the Japanese study would be more clinically relevant, but the study by Das et al. [8] compared the sociodemographic aspect of gerotranscendence.

Although the direction of this link differed significantly, both studies found a statistically significant correlation between gerotranscendence and OHRQoL. According to Mihara et al. [14], there is a positive link between perceived oral health quality and higher levels of gerotranscendence, regardless of objective oral condition. Das et al. [8], on the other hand, reported a negative link and interpreted gerotranscendence as a confounding factor. Higher transcendence may result in a better acceptance of oral restrictions and a lesser level of concern for oral dysfunctions. This apparent discrepancy is probably due to cultural and contextual variations in how spiritual adaptation, contentment, and the personal definition of health in later life are understood.

Older adults with higher levels of gerotranscendence may have greater acceptance and contentment attitudes toward their oral health conditions, which in turn might lead to a self‐reported OHRQoL that reflects this attitudinal threshold, even with objectively poor oral health. This could create a nonclinically significant gap between subjective oral health indicators and clinically significant indicators of oral health in seniors. In this manner, gerotranscendence may better explain the variance between OHRQoL and oral health status and thus represent an important explanatory factor that warrants further exploration into the role of gerotranscendence in older adult perceptions of oral health.

Therefore, the available evidence supports the presence of a relationship between gerotranscendence and OHRQoL but is insufficient to establish its direction, magnitude, or underlying mechanism. Furthermore, as both studies were cross‐sectional, causal interpretations regarding whether gerotranscendence modifies perceptions of oral health or whether oral health experiences influence gerotranscendence cannot be made. These inconsistencies highlight the need for longitudinal and culturally diverse studies using comparable measures of gerotranscendence.

## 4. Discussion

This scoping review centered its results on the relationship between gerotranscendence and OHRQoL among older adults. Two studies were included for the final review, which reveal that the psychosocial component of aging, particularly gerotranscendence, relates to how OHRQoL is perceived by older adults.

The study by Mihara et al. [14] revealed a positive correlation between the GOHAI score and gerotranscendence when controlled for number of teeth, financial status, and occlusal force, which suggests that gerotranscendence among older adults may not be dependent on objective oral health, further emphasizing that gerotranscendence is a psychological facilitator of an individual’s perception of health. Similar results were seen in another study where resilience and coherence have been shown to affect the impact of physical deterioration on quality of life [16].

The study by Das et al. [8] showed contradictory results where there was a negative association between gerotranscendence and GOHAI scores. These differences provoke questions about the role of culture, oral health patterns and behaviors, and psychosocial maturation. A review of studies conducted on certain populations revealed a strong association between their religiousness and happiness, which indicates the influence of culture on their transcendent perceptions [17]. Research on cross‐cultural aging has also suggested that societies that prioritize spirituality and life‐course continuity promote adaptive reconceptualization of health and infirmity [18]. Due to this, individuals may perceive a better quality of life regardless of existing physical impairments. Based on the literature, gerotranscendence depicts a change in the viewpoint from worldly and self‐centric concerns to a meaningful and higher existential reflection and acceptance during the later years of life [19, 20]. This change in psychology may be attributed to a reduction in death anxiety and improved emotional regulation [16, 21, 22]. Research has shown a weak to moderate association between oral health measures and OHRQoL in older adults [23, 24]. It is revealed that several factors such as sense of coherence, resilience, and spirituality facilitate perceptions of health among older adults [25].

Das et al. [8] showed a negative correlation between gerotranscendence and OHRQoL, which could be an indication of how older adults accept and respond to queries regarding health and aging. Research has shown that when transcendent and existential concerns become a priority, there is increased acceptance of decreased body functions and physical constraints in older adults [4, 26]. This could result in individuals responding as having poorer perceived oral health perception even when they exhibit higher gerotranscendence; the reason being that older adults have lower subjective salience in older age, and not due to improved oral health.

Oral health is conventionally associated with esthetics and community participation, and similar consideration may be seen among older adults. Research has shown that tooth loss and edentulism are not clearly accepted among younger adults, whereas older adults reveal better emotional acclimatization and acceptance [27, 28]. This concept is similar to gerotranscendence, where aging is not considered as loss of identity but rather as a part of the normal life transition, which could further describe the occasional disproportionate improvement in OHRQoL after complete rehabilitation among older adults [29].

Studies have shown that lower OHRQoL is correlated with depression, loneliness, and emotional distress, whereas positive outlook and emotional stability are associated with improved health perceptions [7, 30]. Gerotranscendence may promote emotional regulation and reduce anxiety, thus indirectly affecting OHRQoL.

There are certain limitations in this review. First, the number of studies is only two and cross‐sectional in nature, which does not permit causal inference. Longitudinal studies could examine how gerotranscendence develops over time and influences OHRQoL. Second, gray literature was not included in this review, which could have limited the reviewers from finding more evidence. A wider inclusion of various sources may lead to the identification of studies regarding gerotranscendence, OHRQoL, and the factors associated.

## 5. Conclusion

This scoping review shows an intersection between psychosocial aging theory and oral health research. Gerotranscendence seems to modulate the way older adults perceive and respond to challenges presented by their oral health, which suggests that well‐being later in life is not explained by clinical parameters alone. The evidence identified indicates a need to incorporate psychosocial constructs into the models of geriatric oral health and calls for interdisciplinary collaboration between dental researchers, psychologists, and gerontologists.

Geriatric dental assessment should extend beyond clinical findings and include a brief evaluation of patients’ perceived oral health, emotional well‐being, social participation, personal values, and expectations of treatment. Dental professionals can use these insights to support shared decision‐making, provide age‐sensitive communication, and develop treatment plans that prioritize outcomes meaningful to the older person, such as comfort, dignity, functional ability, and social confidence. Whenever psychological concerns arise, a referral pathway involving psychologists, geriatricians, social workers, and other relevant professionals should be established.

## Author Contributions

**Mithun B. H. Pai, Shweta Yellapurkar, Shushma B. Rao, Sampada Suresh Kulkarni, and Kumar Gaurav Chhabra**: conceptualization and design, acquisition, analysis, interpretation of data, drafting or critically revising.

## Funding

This study did not receive any specific funding.

## Disclosure

All authors have read and approved the final version of the manuscript. Shweta Yellapurkar/Mithun Pai B. H. had full access to all the data in this study and takes complete responsibility for the integrity of the data and the accuracy of the data analysis.

## Ethics Statement

Ethical approval or consent to participate is not applicable for this scoping review, as it synthesizes existing published literature and does not include primary data collection from human participants.

## Conflicts of Interest

The authors declare no conflicts of interest.

## Supporting Information

Additional supporting information can be found online in the Supporting Information section.

## Supporting information


**Supporting Information** Supporting Table: Methodological quality appraisal of the included studies using the JBI Critical Appraisal Checklist for Analytical Cross‐Sectional Studies, of Mihara et al. [14] and Das et al. [8].

## Data Availability

The authors affirm that the data that support the results from this study are accessible in the article and/or in the Supporting Information section. The current study did not generate or analyze datasets.
