# Supplementary material for: Beyond Aging: The Relationship Between Gerotranscendence and Oral Health‐Related Quality of Life—A Scoping Review
Source: Int J Dent. 2026 Oct 13;2026:3541650. doi: 10.1155/ijod/3541650 (PMC13662413; doi:10.1155/ijod/3541650)
Supplement: Supplementary file 1 [file IJOD-2026-3541650-s001.docx]

| **JBI Critical Appraisal Criterion** | **Das et al., 2023** | **Mihara et al., 2018** |
| --- | --- | --- |
| 1. Were the criteria for inclusion in the sample clearly defined? | **Yes** | **Yes** |
| 2. Were the study subjects and the setting described in detail? | **Yes** | **Yes** |
| 3. Was the exposure measured in a valid and reliable way? | **Yes** | **Yes** |
| 4. Were objective, standard criteria used for measurement of the condition? | **Yes** | **Yes** |
| 5. Were confounding factors identified? | **Yes** | **Yes** |
| 6. Were strategies to deal with confounding factors stated? | **Yes** | **Yes** |
| 7. Were the outcomes measured in a valid and reliable way? | **Yes** | **Yes** |
| 8. Was appropriate statistical analysis used? | **Yes** | **Yes** |

**Table: Methodological quality appraisal of the included studies using the JBI Critical Appraisal Checklist for Analytical Cross-Sectional Studies**

*JBI: Joanna Briggs Institute; Yes = criterion adequately addressed; No = criterion not addressed; Unclear = insufficient information reported; N/A = not applicable.*
